# Fritz Köberle: A Life Dedicated to Medicine

**DOI:** 10.1590/0037-8682-0045-2025

**Published:** 2025-03-31

**Authors:** Luiz Eduardo Correia Miranda

**Affiliations:** 1 Universidade de Pernambuco, Hospital Universitário Oswaldo Cruz, Recife, PE, Brasil.

Fritz Köberle, an Austrian-Brazilian physician and scientist born on October 1, 1910, in Eichgraben, Austria, is renowned for his contributions to the understanding of Chagas disease. The tumultuous socio-political landscape of the 20th century had a profound effect on Köberle's life and career.

Köberle's early life was rooted in the idyllic traditions of agriculture and close-knit community life set against the backdrop of the crumbling Austro-Hungarian Empire. The early 20th century bore witness to rising nationalist sentiment, social inequalities, and the burgeoning influence of socialist and liberal ideologies, resulting in an atmosphere of political instability. The outbreak of World War I in 1914 accelerated the disintegration of the empire, culminating in its collapse in 1918. These formative years influenced Köberle's worldview^1^. 

Köberle's education, spanning from elementary school to university, was completed entirely in Vienna. After graduating with honors from the Humanistic Gymnasium in Vienna, he pursued medicine at the University of Vienna. The University of Vienna, founded on March 12, 1365, by Duke Rudolph IV of Austria, is one of Europe's oldest and most prestigious institutions for higher learning. The university, known as Alma Mater Rudolphina, has played a vital role in educating generations of thinkers, scientists, and artists across several fields of knowledge for over 650 years. Köberle's early career following his university studies was characterized by a strong interest in research and the pursuit of new knowledge. His academic career in pathology was commenced at the Vienna Polyclinic^2^.

The rise of Nazism in Europe altered the course of Köberle's life, as a Jewish man. He was persecuted and compelled to serve in the German army following the annexation of Austria by Germany in 1938. His military service took him to the Eastern Front where he served as a pathologist. The specifics of his life on the Eastern Front remain largely unknown; however, a bout of malaria led to his return from combat.

Köberle held various positions in Münster and Vienna following the war. Notably, he accepted an invitation from Professor Zeferino Vaz, in 1953 to head the Department of Pathology at the newly inaugurated Ribeirão Preto Medical School, University of São Paulo (USP), Brazil, marking a turning point in his career and a new chapter in his life. Köberle, who had previously visited Brazil in 1938, embraced the opportunity to contribute to the development of medicine in his adopted country^2^.

Köberle's arrival in Ribeirão Preto coincided with the opportunity to study Chagas disease. He noted a high prevalence of megaesophagus, a rare manifestation of this disease that was rarely observed in Europe, eliciting his interest in the study of the pathology of Chagas disease. Köberle built upon the work of Carlos Chagas, who described the complete cycle of Chagas disease in 1909. Köberle elucidated the neurogenic mechanism of the chronic phase of Chagas disease by demonstrating denervation of the heart and other organs induced by *Trypanosoma cruzi*. Thus, his contributions to the understanding of infection-associated cardiac alterations are seminal.

A curious anecdote was recounted by Professor João Samuel de Oliveira, a professor of pathology at the Ribeirão Preto School of Medicine who worked directly with Professor Köerbele. Early in his career in Ribeirão Preto, Köerbele was surprised to discover a megaesophagus during one of the first autopsies he performed. Given that such a specimen was rarely encountered in Europe, he considered it a stroke of good fortune and anticipated valuable publications from its study. The discovery of another megaesophagus during another autopsy conducted within days prompted further investigation. A third case was encountered a few days later. Köerbele, who was incredulous, believed that something was amiss. Noticing the esteemed professor's bewilderment, a pathology technician explained, “That's Chagas disease, Doctor. It's quite common here.” Although familiar with Chagas disease from discussions with Professor Rocha Lima before his arrival in Brazil, Koerbele had never encountered a case. Thus, he dedicated himself to the study of the pathology of the disease, capitalizing on the high prevalence in the region^2^, as its pathology remained poorly understood^3^ despite Dr. Carlos Chagas describing the complete disease cycle in 1909.

Köerbele hypothesized that the lesions observed in Chagas disease result from motor alterations owing to the destruction of the myenteric plexus, a concept previously put forth by Brazilian researchers. This finding contradicts the prevailing hypothesis that a parasite present within the muscular wall of the affected organ causes the disease. Köeberle quantified myenteric plexus neurons in standardized histological sections of diseased organs and compared them with samples from healthy individuals and patients with Chagas disease without gastrointestinal manifestations. Few neurons were retained amid the inflammatory process, or the plexus was replaced by fibrous scar tissue in patients with megaesophagus exhibiting significant organ dilation. Therefore, Köeberle characterized Chagas disease as a disorder of the autonomic nervous system^4^ ^,^ ^5^.

Koeberle's denervation theory of the "megas" was met with resistance within the academic community^6^. Some researchers proposed a viral etiology. However, Koeberle humorously retorted, "Only if the virus comes mounted on a trypanosome"^2^_._

Köberle's influence extends beyond the scope of this study. He trained generations of physicians and researchers who became leaders in pathology in Brazil as a professor at USP. He held several prominent positions, including Professor of Pathology and Director of the Department of Pathology at USP. He also served as President of the Brazilian Society of Pathology and was a member of numerous national and international scientific societies.

Köberle's prolific contribution includes numerous articles published in national and international journals, as well as books and book chapters on pathology and tropical diseases, which have earned him international recognition and numerous awards^2^ ^,^ ^7^. His work is an essential part of the curriculum for students and researchers.

Fritz Köberle died in Americana, São Paulo, Brazil on February 20, 1983, leaving behind a legacy of groundbreaking research and dedicated mentorship that significantly advanced the understanding and treatment of Chagas disease.
